# Exosomal miR-665 as a novel minimally invasive biomarker for hepatocellular carcinoma diagnosis and prognosis

**DOI:** 10.18632/oncotarget.20881

**Published:** 2017-09-14

**Authors:** Zhen Qu, Junhua Wu, Junyi Wu, Anlai Ji, Guanghui Qiang, Yong Jiang, Chunping Jiang, Yitao Ding

**Affiliations:** ^1^ Department of Hepatobiliary Surgery, Drum Tower Hospital, Medical School of Nanjing University, Nanjing, Jiangsu Province, China; ^2^ Jiangsu Key Laboratory of Molecular Medicine, Medical School of Nanjing University, Nanjing, Jiangsu Province, China; ^3^ Department of Hepatobiliary Surgery, The First People's Hospital of Changzhou, The Third Hospital Affiliated to Soochow University, Changzhou, Jiangsu, China; ^4^ Department of General Surgery, The Affiliated Hospital of Yangzhou University, Yangzhou, Jiangsu, China; ^5^ Department of Hepatobiliary Surgery, Drum Tower Clinical College of Nanjing Medical University, Nanjing, Jiangsu Province, China

**Keywords:** HCC, exosomal miRNA-665, biomarker, ERK, tumor growth

## Abstract

Recent studies have shown that circulating microRNAs are potential biomarkers for various types of malignancies. The aim of this study was to investigate the feasibility of using serum exosomal microRNAs (miRNAs) as novel serological biomarkers for hepatocellular carcinoma (HCC) diagnosis and prognosis. Exosomes are small membranous vesicles (30–100 nm). Exosomal miR-665 levels in HCC patients were significantly higher than those in healthy subjects (*P* < 0.05), and exosomal miR-665 levels were significantly upregulated in tumours larger in size (> 5 cm), in tumours with local invasion and in those at an advanced clinical stage (stage III/IV) of HCC (*P* = 0.0042, 0.0197, and 0.0276, respectively). The survival time of the exosomal miR-665 high-expression group (*n* = 17) was significantly shorter than that of the low-expression group (*n* = 13) (*P* = 0.036). In addition, we found that HCC cell-derived exosomes promoted hepatoma cell proliferation and upregulated the expression level of proteins in the MAPK/ERK pathway *in vitro* and *in vivo*. This study suggests that serum exosomal miR-665 may be a novel minimally invasive biomarker for HCC diagnosis and prognosis.

## INTRODUCTION

Hepatocellular carcinoma (HCC) is the second highest cause of tumour-related death worldwide [1]. The incidence of male and female HCC cases in China is the highest in the world. Currently, a surgical-based integrated treatment model is employed for HCC treatment, but most patients are not eligible for surgery at diagnosis. Combined with the high invasion and metastasis degree of HCC, the prognosis of HCC patients is extremely poor [2, 3]. Therefore, it is of great significance to identify sensitive and specific markers for the early diagnosis of HCC.

MicroRNA (miRNA) is a class of noncoding small RNA approximately 19–25 bp long that can function at the 3′ non-coding region (3′ untranslated region; 3′UTR) of mRNA through fully complementary or incomplete complementary means, achieving the negative regulation of genes at the post-transcriptional level by degrading mRNA or inhibiting the translation of target genes [4]. Accumulating evidence indicates that miRNAs are involved in the proliferation, metastasis and angiogenesis of tumour cells [5]. miRNAs are highly expressed in the serum of patients with many types of tumours, such as HCC [6], colorectal cancer [7], and pancreatic cancer [8], suggesting that miRNA expression in serum may be a new non-invasive marker for the diagnosis of multiple tumours, including HCC.

Exosomes are nanocapsule vesicles secreted by many types of cells during physiological processes and contain specific biological factors, such as miRNAs, mRNAs and proteins [9]. Exosomes can be extracted from a variety of biological fluids, including serum, urine, and ascites [10, 11]. Reports have indicated that serum miRNA is mainly contained in exosomes to avoid degradation by RNAase [12]. Therefore, exosomal miRNAs can be effective, non-invasive biological markers for disease diagnosis and prognosis determination [13–15]. However, there have been few studies exploring the value of exosomal miRNAs in the diagnosis and prognostic evaluation of HCC or the mechanism of specific actions of miRNA on HCC cells.

In this study, we hypothesized that exosomes from different sources contain different levels of miRNAs, which likely differentially affect HCC tumour progression. Therefore, through the evaluation of the differential expression of miRNAs in exosomes derived from hepatomas with different degrees of malignancy identified by microarray, miR-665 was identified as a miRNA with significantly differential expression in different groups. To date, there have been few reports regarding the role of miR-665 in tumours [16–18], and its role in HCC has not been reported. By detecting miR-665 expression levels in exosomes separated from the serum of HCC patients and healthy controls, we further explore the value of exosomal miR-665 in the early diagnosis and prognosis of HCC. In addition, *in vitro* and *in vivo* experiments were performed to explore the proliferative effect of exosomal miR-665 HCC cells and its potential mechanism, with the goal of facilitating the early diagnosis and prognosis prediction of HCC.

## RESULTS

### Characterization of isolated exosomes

**Figure 1:**
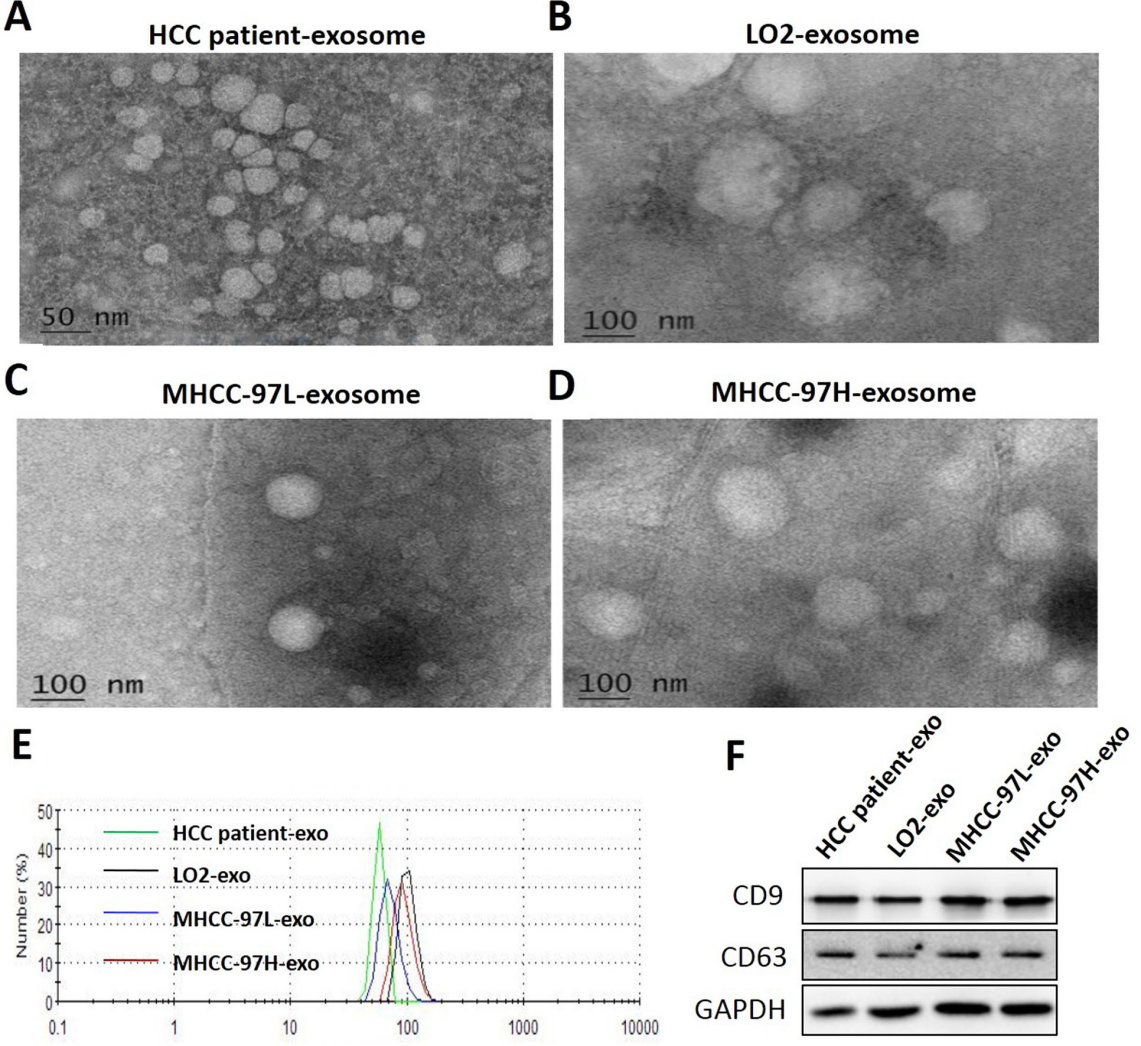
Characterization of isolated exosomes Exosomes were purified from the serum of HCC patients (**A**), LO2 (**B**), MHCC-97L (**C**) and MHCC-97H (**D**) cell culture supernatant and examined by TEM. (**E**) Size distribution analysis of purified exosomes using a Nanosight instrument. (**F**) Exosome markers (CD9, CD63) were analysed using Western blot. The data demonstrated that extracts were enriched with exosomal marker proteins CD9 and CD63.

To ensure the efficacy and quality of the exosomes isolated from serum and cell culture supernatants, we characterized the microvesicles by TEM, nanosight and Western blot analysis. Electron microscopic analysis of the exosomes isolated from serum and cell culture supernatant samples showed circular structures with sizes varying between 50 and 150 nm (Figure 1A–1E), consistent with previously reported characteristics of exosomes. Further, the identification of exosomes was confirmed by the detection of the specific exosomal protein markers CD9 and CD63 and using Western blot analysis (Figure 1F). These results confirmed the successful isolation of exosomes from different samples.

### Differential expression of miRNA in hepatoma cell exosomes

**Figure 2:**
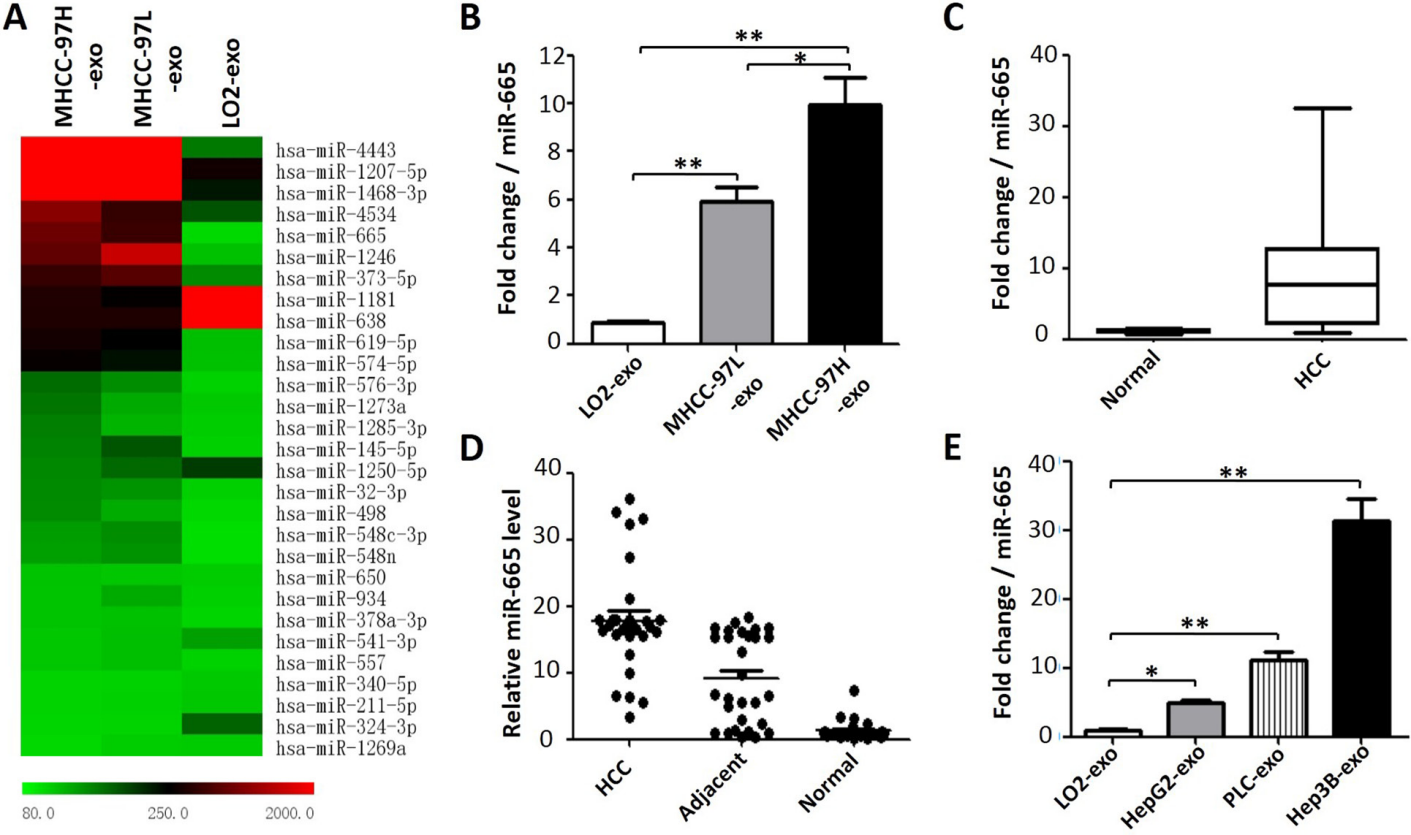
Exosomal miR-665 expression is significantly higher in HCC (**A**) A heatmap representing the differential miRNA expression in exosomes derived from three cell lines. The colour “red” indicates high relative expression, and “green” indicates low relative expression. (**B**) RT-PCR validated the reliability of the miRNA microarray, the average fold increase of exosomal miR-665 in MHCC-97H and MHCC-97L cells vs. the normal liver cell line (LO2) were 10.1 and 5.8, respectively. *P < 0.05; **P < 0.01. (**C**) The levels of exosomal miR-665 in serum samples from 30 patients with HCC and 10 healthy controls were determined by RT-PCR. (**D**) miR-665 expression in HCC, adjacent and normal liver tissue. The absolute 2^−ΔΔCT^ values are presented. (**E**) miR-665 expression was detected by RT-PCR in the exosomes derived from multiple hepatoma cell lines (HepG2, PLC and Hep3B).

First, we used miRNA microarray to screen the differentially expressed miRNAs in three exosomes isolated from cell culture supernatants of HCC samples with different degrees of malignancy. In this study, differentially expressed miRNAs were selected according to the following criteria: the fold change was no less than 1.5 for MHCC-97H exosomes vs. MHCC-97L exosomes; the fold change was no less than 5 for MHCC-97L exosomes vs. LO2 exosomes; and the *P* value was set at 0.05. The results suggested that MHCC-97H and MHCC-97L exosomal miR-665 expression was higher than that in LO2 cell lines (Figure 2A). The average fold increases of miR-665 in MHCC-97H and MHCC-97L compared to the levels in the normal liver cell line were 10.1 and 5.8, respectively. RT-PCR validated the miRNA microarray results (Figure 2B). In addition, we also detected the miR-665 expression in exosomes from multiple hepatoma cell lines (HepG2, PLC and Hep3B). We found that the exosomal miR-665 expression was significantly upregulated in these three hepatoma cell lines compared with that in normal liver cells (Figure 2E).

### Clinical significance of exosomal miR-665 in HCC

To understand the potential value of serum exosomal miR-665 in HCC development and progression, the expression levels of exosomal miR-665 in 30 patients with HCC and 10 healthy individuals in the follow-up cohort were determined using qRT-PCR. As expected, the expression levels of exosomal miR-665 in the HCC group were significantly increased compared with the control group (Figure 2C). Moreover, we also detected the miR-665 expression in 30 pairs of HCC tissue and adjacent tissue, and the results were similar to the previous serum data. We found that miR-665 expression in HCC tissue was substantially higher than that in adjacent tissue and normal liver tissue (Figure 2D).

**Figure 3:**
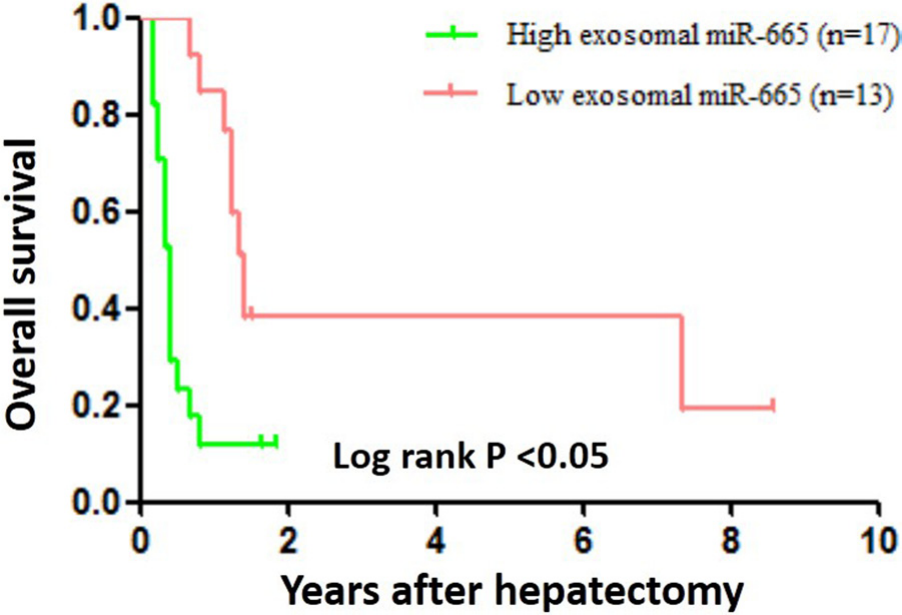
Overall survival curves for 30 HCC patients who underwent hepatectomy The patients with high exosomal miR-665 expression exhibited significantly poorer long-term prognosis after hepatectomy.

To better understand the potential roles of serum exosomal miR-665 in HCC development and progression, we validated the clinicopathological significance of miR-665 expression in serum exosomes by RT-PCR. We categorized 30 HCC cases into two groups according to the mean miR-665 expression level in preoperative serum exosomes. In this study, a 5-fold increase was used as the cut-off value to divide the samples into high-miR-665-expression (≥ 5-fold) and low-miR-665-expression (< 5-fold) groups. The statistical analysis revealed that, compared with the low-miR-665-expression group, the high-expression group showed higher clinical stages (*P* = 0.0276) and stronger tendencies of association with larger tumour size (> 5 cm; *P* = 0.0042) and local tumour invasion and metastases (*P* = 0.0197, Table 1). However, there was no correlation of exosomal miR-665 expression with other clinical features, including age, sex, HBV infection and AFP level (*P* > 0.05). Furthermore, we examined the association between serum exosomal miR-665 expression and HCC patient prognosis using 30 primary HCCs with hepatectomy. Patients with exosomal miR-665 expression levels less than the 5-fold were assigned to a low-expression group (*n* = 13) and those above 5-fold to a high-expression group (*n* = 17). Further investigation demonstrated that the high-miR-665-expression group had a significantly poorer prognosis than the low-expression group (*P* < 0.05, Figure 3).

**Table 1:** Correlations of patient clinicopathologic characteristics with exosomal miR-665 expression level

Clinicopathological features			Exosomal miR-665		*P* value
			Low *n*(%)	High *n*(%)	
Sex	F	18	8 (44.4)	10 (55.6)	
	M	12	5 (41.7)	7 (58.3)	*P* = 0.8804
Age	> 60	12	5 (41.7)	7 (58.3)	
	≤ 60	18	8 (44.4)	10 (55.6)	*P* = 0. 8804
HBsAg	+	24	11 (45.8)	13 (54.2)	
	–	6	2 (33.3)	4 (66.7)	*P* = 0.5805
AFP (ng/mL)	> 20	22	10 (45.5)	12 (54.5)	
	≤ 20	8	3 (37.5)	5 (62.5)	*P* = 0.6974
Cirrhosis	+	20	8 (40.0)	12 (60.0)	
	-	10	5 (50.0)	5 (50.0)	*P* = 0.6023
Tumour size (cm)	> 5	20	5 (25.0)	15 (75.0)	
	≤ 5	10	8 (80.0)	2 (20.0)	*P* = 0.0042
Capsule invasion	+	9	1 (11.1)	8 (88.9)	
	–	21	12 (57.1)	9 (42.9)	*P* = 0.0197
TNM stage	III–IV	24	8 (33.3)	16 (66.7)	
	I–II	6	5 (83.3)	1 (16.7)	*P* = 0.0276

### Exosomal miR-665 promotes HCC cell proliferation

**Figure 4:**
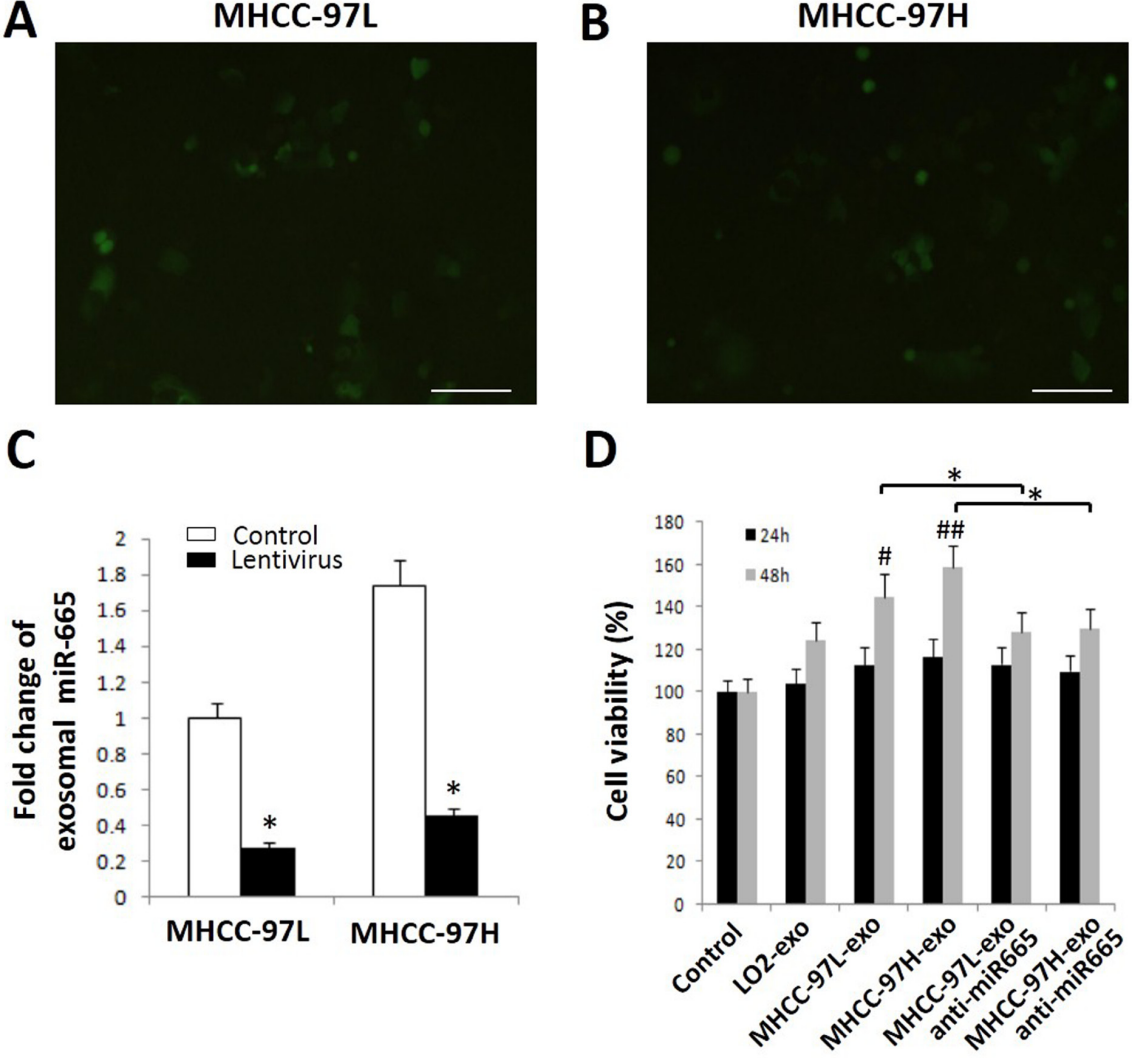
Exosomal miR-665 promotes cell proliferation in HCC cells Micrographs of MHCC-97L (**A**) and MHCC-97H cells (**B**) infected with lentivirus-green fluorescent protein, captured after 72 h under a fluorescence microscope. Scale bar represents 500 μm. (**C**) miR-665 expression in the lentivirus group and control group. *P < 0.05. (**D**) Cell viability was assessed using an MTT assay. HCC exosomes promote cell proliferation in HCC cells; after the application of anti-miR-665, the cell proliferation-promoting effects of exosomes were downregulated. Equal volume of PBS was used as a control. *P < 0.05.

Because high exosomal miR-665 expression was associated with the malignant potential of HCC, the function of exosomal miR-665 was assessed using an MTT assay. The MTT results showed that HCC-derived exosomes promoted the proliferation of SMMC-7721 cells in a time-dependent manner, and exosomes derived from tumour cells with higher exosomal miR-665 levels had greater efficacy. After the application of anti-miR-665, the cell proliferation-promoting effects of exosomes were suppressed (*P* < 0.05, Figure 4).

### Exosomal miR-665 affects cell proliferation and tumour growth through the MAPK/ERK pathway

**Figure 5:**
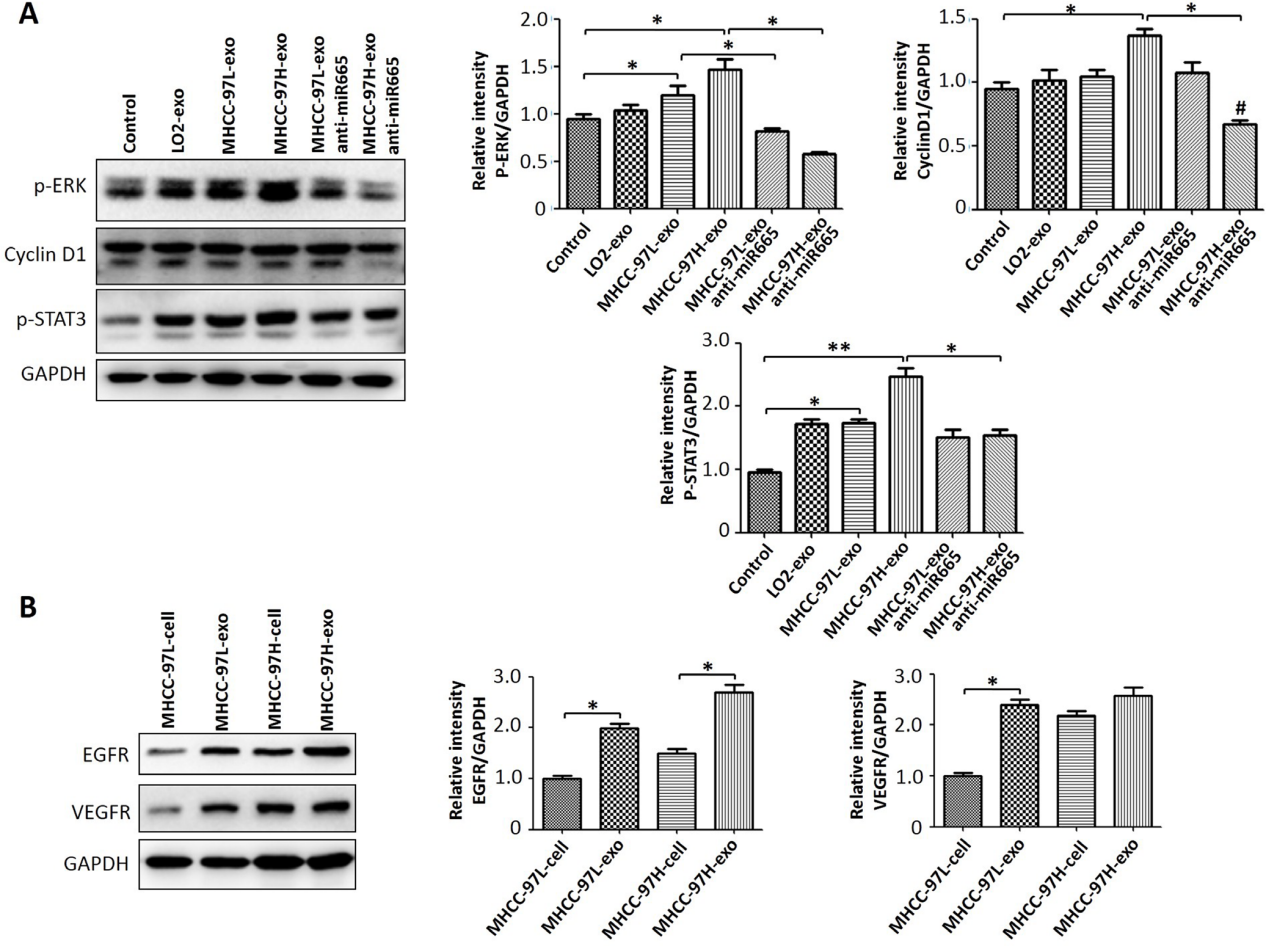
Exosomal miR-665 upregulated the expression of HCC cell proliferation-related proteins (**A**) The expression levels of p-ERK, CyclinD1 and p-STAT3 were detected by Western blot; an equal volume of PBS was used as a control. Exosomal miR-665 could promote HCC cell proliferation through activating the MAPK/ERK pathway; the effects of exosomes decreased with the application of anti-miR-665 in HCC cells. (**B**) The expression levels of VEGFR and EGFR were detected by Western blot. HCC-derived exosomes contained many different molecules (VEGFR and EGFR). *P < 0.05.

To investigate whether the effect of exosomal miR-665 on HCC cell proliferation was related to the activation of ERK and other proteins, we detected the expression levels of p-ERK, p-STAT3, and EGFR in SMMC-7721 cells using Western blotting. The results showed that exosomes could upregulate the expression of p-ERK, and MHCC97H-derived exosomes caused the most significant effects (Figure 5A). In addition, HCC-derived exosomes contained many different molecules (Figure 5B). These results suggested that HCC-derived exosomes may also transmit these factors to influence the microenvironment.

**Figure 6:**
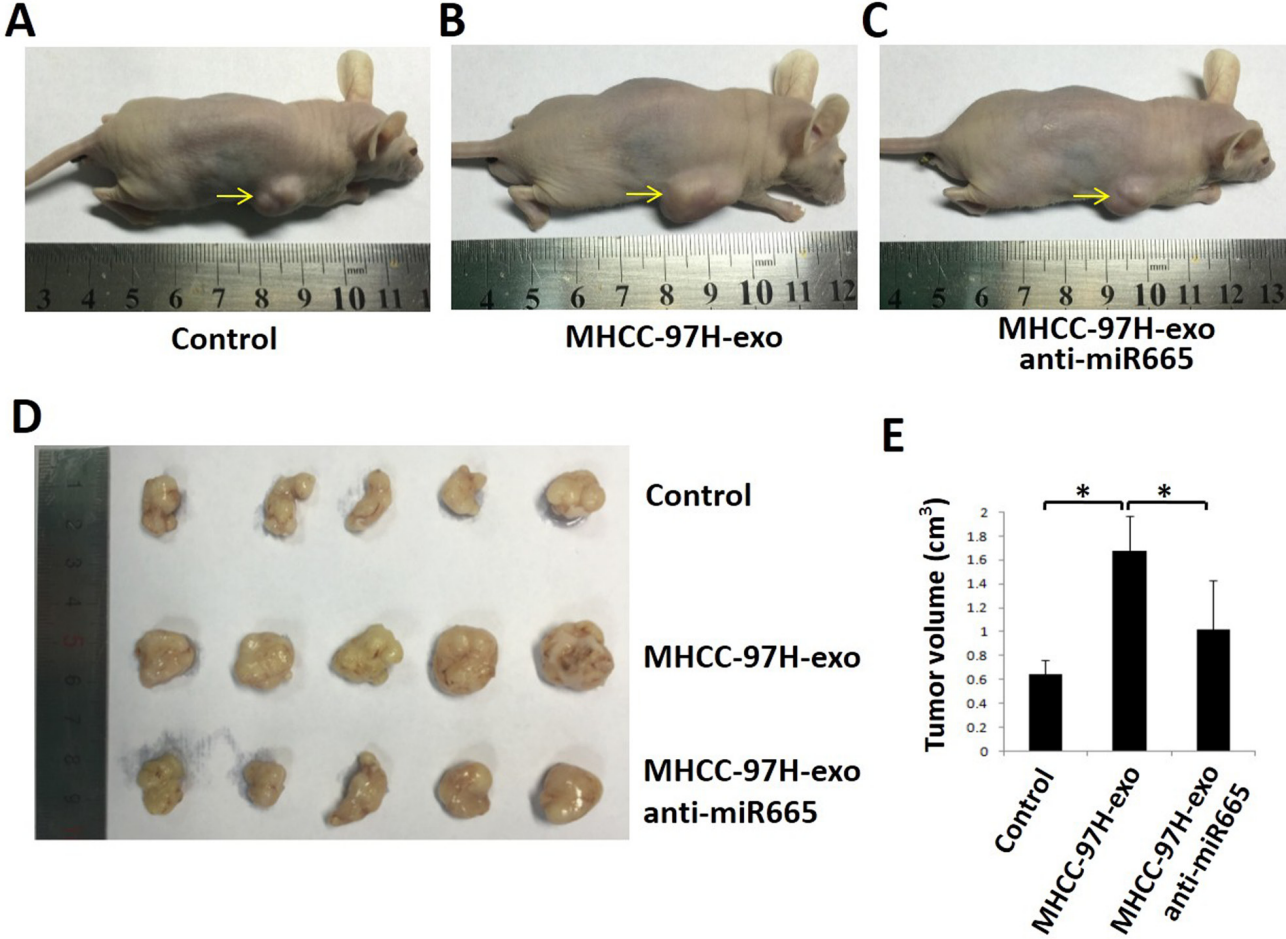
Exosomal miR-665 promoted xenograft tumour growth *in vivo* The size of tumours at the end of the experiment from mice treated with PBS (Control, **A**), MHCC97H-exosome (**B**) and MHCC97H-exosome anti-miR-665 (**C, D, E**) Representative images and tumour volume changes in the mice bearing MHCC97H-exosome or MHCC97H- exosome anti-miR-665. *n* = 5, significant difference between groups are shown as *P < 0.05.

**Figure 7:**
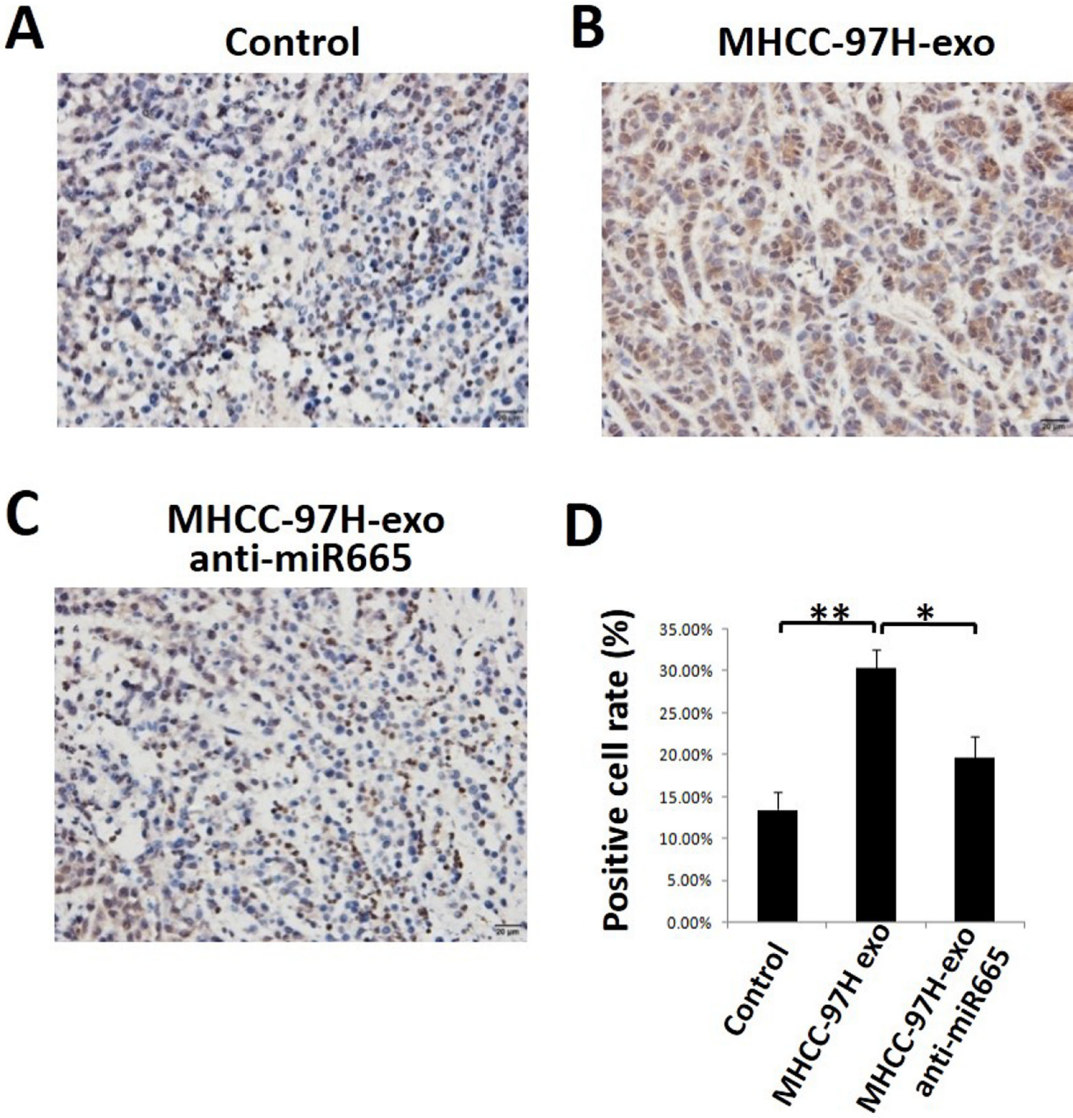
MHCC97H-exosome anti miR-665 downregulated the expression of P-ERK *in vivo* Tumours from mice treated with PBS (Control, **A**), MHCC97H-exosome (**B**) and MHCC97H-exosome anti-miR-665 (**C**) were paraffin-embedded and sectioned, followed by staining by using immunohistochemistry method. (**D**) The number of P-ERK-positive cells notably decreased in the MHCC97H-exosome anti-miR-665 group compared with the number in the MHCC97H-exosome group. *P < 0.05.

To detect whether exosomal miR-665 could promote tumour growth in liver cancer *in vivo*, we established a subcutaneous xenograft model in nude mice and injected MHCC-97H-derived exosomes or MHCC-97H exosome-anti-miR-665 into mouse subcutaneous tumours. As shown in Figure 6, the tumours in mice treated with MHCC-97H-derived exosomes were significantly larger than control groups; this effect was obviously weakened in the exosome-anti-miR-665 group, indicating that exosomal miR-665 could promote HCC growth and progression. Figure 6E shows the tumour volume of each group; it is clear that the tumour volume in mice treated with MHCC-97H-exosomes was significantly greater than that in other groups. Furthermore, we detected the expression of p-ERK in mouse subcutaneous tumours using immunohistochemistry. The results showed that MHCC-97H-exosomes upregulated the expression of p-ERK, and the knock-down of exosomal miR-665 expression obviously weakened this effect (Figure 7).

## DISCUSSION

Currently, the clinical diagnosis of HCC depends on AFP and imaging examination, such as colour Doppler ultrasound, computed tomography (CT), magnetic resonance imaging (MRI), and tumour cytological biopsy. However, an early diagnosis of HCC is still difficult [19], and many patients are not eligible for surgery at diagnosis. Therefore, the identification of new and sensitive biomarkers for the early diagnosis of HCC is urgently needed.

Many studies have confirmed that miRNAs are involved in tumour formation and development [20, 21]. Studies have suggested that serum miR-185 can not only be used as a marker for early-stage HCC but is also closely related to liver metastasis and tumour size [22]. However, the molecular mechanism and function of most HCC-related miRNAs remain unclear. Exosomes derived from different tumours contain miRNA with specific characteristics. Recipient cells demonstrate obvious tumourigenic tendencies after cellular uptake of such miRNAs, including tumour cell migration, immune response, angiogenesis, and the infiltration and metastasis of tumour cells [23, 24]. A previous study found that the levels of miR-21 and miR-146a in exosomes derived from cervical cancer were significantly elevated [25]. Liu et al utilized diethylnitrosamine to induce the establishment of a rat HCC model and found that miRNAs of the combined cycle (miRNA-10b, miRNA-21, miRNA-122, and miRNA-200a) and exosomes can be used to effectively diagnose early-stage HCC [26]. Xiang et al found that tumour-derived exosomal miR-222-3p is an effective regulator in the polarization of tumours and may be a biomarker of epithelial ovarian cancer [27]. Another study showed that the serum exosomal miRNAs of patients might be used as effective biomarkers in predicting HCC recurrence through the detection of serum exosomal miRNA levels in liver transplantation patients. It was found that exosomal miR-718 level is associated with tumour recurrence, metastasis and prognosis [28]. In summary, understanding the functions of miRNAs with abnormal expression in HCC and their related mechanisms, especially the identification of miRNAs with abnormal expression, by high-throughput sequencing screening is particularly important, and this information may improve our understanding of the development mechanisms of HCC and provide new targets for HCC treatment.

Previous studies are often limited to *in vitro* or animal experiments, while the mechanism of action and clinical evidence for the role of exosomes and miRNA remain unclear. The current study utilized a miRNA microarray chip to identify exosomal miRNAs with significantly different expression levels in different hepatoma cell lines and found that exosomal miR-665 derived from different cells showed significantly different expression levels. qRT-PCR validation found that the exosomal miR-665 levels in MHCC-97H cells with a high degree of malignancy were 2- and 10-fold those in MHCC-97L and LO2 cells, respectively. In addition, qRT-PCR was used to detect serum exosomal miR-665 levels in clinical HCC patients and normal controls and found that the exosomal miR-665 levels in the serum of HCC patients was significantly higher than that of the healthy control group. Furthermore, the exosomal miR-665 level closely correlated with the clinicopathological parameters of patients, such as clinical stage, tumour differentiation, and patient survival, suggesting that serum exosomal miR-665 may be extensively involved in HCC occurrence and development. In addition, previous studies have found that exosomes may transmit VEGF and miRNAs and promote tumour progression and metastasis (by affecting cell phenotype changes, etc.), suggesting that exosomes and key miRNAs are closely involved in tumour progression and may be a potential tumour therapeutic target [29, 30].

Skog et al [31] reported that exosomes released by primary cultured malignant glioma cells could promote the proliferation of human glioma cells. An *in vivo* study also confirmed that exosomes derived from mouse breast cancer cells could inhibit the function of NK cells and promote tumour growth [32]. The research of Takayuki et al showed that exosome-mediated miRNA transport play an important role in the communication process among HCC cells, promoting tumour growth and metastasis, the mechanism of which may be related to regulation of transforming growth factor B activated kinase-1 (TAK1) expression by exosomes derived from HCC cells and the related signalling pathways [24]. These results suggest that tumour-derived exosomes play an important role in the process of tumour cell proliferation and metastasis. Our study found that exosomes derived from different HCC cell lines could promote the proliferation of the HCC cell line SMMC-7721 in a co-culture. The MAPK/ERK signal pathway is an important intracellular proliferative and anti-apoptotic pathway and plays an important role in the malignant proliferation of tumour cells by affecting the activity of effector molecules, such as downstream cell cycle regulatory proteins and apoptosis-related proteins [33, 34]. Recent studies have indicated that MAPK/ERK signalling molecules are overexpressed or consistently activated in gastric cancer [35], colon cancer, breast cancer [36, 37], and other tumours. Our results showed that the exosomes of HCC cells activated the MAPK/ERK pathway when promoting the proliferation of SMMC-7721 cells, and the expression of proteins in the MAPK/ERK pathway was reduced when anti-miR-665 was introduced, suggesting that tumour-derived exosomes may activate the MAPK/ERK pathway through miR-665 and further promote the proliferation of tumour cells. In addition, the analysis of exosomal protein showed that it contained VEGF, which was closely related to the malignant proliferation of tumour cells. By increasing the expression of growth factor surface receptors through binding with tumour cell surface receptors or directly fusing with the cell membrane, the expression of these molecules is likely to play a role in promoting proliferation.

Although the physiological role of tumour-cell-derived exosomal miR-665 remains unclear, it is certain that exosomal miR-665 plays an important role in the *in vivo* growth and development of tumour cells. The results of the current study showed that tumour-originated exosomal miR-665 could promote tumour cell proliferation *in vitro*, and the mechanism may be related to activation of the MAPK/ERK pathway by biological factors inside the exosomes, such as VEGF. Because exosomes are bioactive structures actively produced and secreted by living cells, they contain a variety of biological factors, including mRNAs and miRNAs, and demonstrate complicated and changeable functions [38]. Therefore, an in-depth understanding of the molecular mechanisms of tumour-derived exosomes in promoting HCC cell proliferation has important significance for clarifying of the role of exosomes in the occurrence and development of HCC and their potential therapeutic value.

## MATERIALS AND METHODS

### Cells and reagents

MHCC-97H, MHCC-97L and LO2 cell lines were obtained from the Liver Cancer Institute, Fudan University (Shanghai, China). SMMC-7721 cell line was purchased from Cell Bank of Xiangya Central Experiment Laboratory of Central South University (Changsha, China). MHCC-97H, MHCC-97L and SMMC-7721 were maintained in Dulbecco's Modified Eagle Medium (DMEM, WISENT, CA, USA) containing 10% foetal bovine serum (FBS) (ExCell Bio, China). LO2 cell line was maintained in 1640 containing 10% foetal bovine serum (FBS) (ExCell Bio, China). All cells were incubated in 5% CO_2_ at 37°C. MTT (3-(4,5-dimethylthiazol) 2, 5-diphenyltetrazolium) was purchased from Sigma Aldrich (St. Louis, MO, USA). The antibodies for glyceraldehyde-3-phosphate dehydrogenase (GAPDH), p-Akt, Akt, p-ERK, p-JNK, p-P38 and EGFR were obtained from Cell Signaling Technology (Beverly, MA, USA). The antibody for VEGFR-2 was obtained from Bioworld Technology Inc. (Bioworld, USA).

### Patient samples

The thirty serum specimens and HCC tissue specimens were collected from HCC patients in the Department of Hepatobiliary Surgery of the Affiliated Drum Tower Hospital, Medicine School of Nanjing University (Nanjing, China). No patients received any anti-cancer treatment or had any other endocrine, immune, or metabolic diseases. The tumour type and the grade of cell differentiation were diagnosed based on the criteria of the World Health Organization (WHO), whereas the pathological stage of each tumour was determined by the International Union Against Cancer (UICC) TNM classification. The normal control group contained ten samples from healthy people. Written informed consent was obtained from all subjects prior to the recruitment. The study protocol was approved by the Institutional Review Board of the hospital ethics committee. The clinical characteristics of the subjects are listed in Table 2.

**Table 2:** Clinical characteristics of the subjects (n = 40)

Variables: median (range)	Healthy control (*n*= 10)	HCC (*n* = 30)	*P*-value
Sex (M/F)	6/4	18/12	*P* = 1.0
Age (years)	53.0 ± 2.2	54.3 ± 2.4	*P* = 0.36
ALT	27.4	38.5	*P* = 0.031
AST	25.7	40.2	*P* = 0.026
AFP (ng/ml)	2.8	1083.8	*P* < 0.001
HBsAg-positive	2	24	*P* < 0.001
Tumour size (cm)		4.5	
Tumour number (1/2/3 ∼)		21/5/4	
Capsule invasion (+/−)		9/21	
TNM stage (I–II/III–IV)		6/24	

### Isolation of exosomes

Peripheral blood was collected and centrifuged at 3,000 rpm for 10 min at 4°C to spin down the blood cells. The supernatants were centrifuged at 12,000 g for 10 min at 4°C to completely remove the cellular components. The serum samples were stored at −80°C until use. Exosomes were isolated from serum samples using Exoquick TM Kit (System Biosciences, USA) following the manufacturer's protocol. Briefly, 0.1 mL of Total Exoquick TM Reagent was added to 0.4 mL of serum and incubated for 12 h at 4°C, followed by centrifugation at 10,000×g for 30 min at room temperature. Finally, the exosome pellets were collected for characterizations and RNA extractions.

Different cell lines were cultured in media with 10% exosome-free FBS (by ultracentrifugation overnight). After 48 h, cell culture media were collected, and exosomes were isolated from the supernatant by differential centrifugation as previously described (see “Isolation of exosomes”) [39].

### Transmission electron microscopy

The extracted pellets were observed under transmission electron microscopy (TEM) as previously described. A drop of purified exosomes (approximately 10 μL) was fixed with 1% glutaraldehyde for 10 min, washed, and contrasted in 2% uranyl acetate. Images were obtained by TEM (JEM-2100, Jeol, Japan), and the size distribution of the isolated exosomes was analysed by Nanosight instrument (Malvern, UK) according to the manufacturer's instructions.

### Western blot analysis

To determine the level of indicated proteins, exosomes and hepatoma cells were lysed with RIPA peptide lysis buffer (Beyotime Biotechnology, China) containing 1% protease inhibitors (Pierce). Twenty micrograms of proteins was analysed by Western blot as described. The PVDF membranes with transferred proteins were incubated with primary antibodies at 4°C overnight and HRP-conjugated secondary antibodies at room temperature for 2 h. The signal was developed by the enhanced chemiluminescence (ECL) reagent (Millipore, Bedford, MA, USA) and visualized by FluorChem FC2 Imaging System (Alpha Innotech, San Leandro, CA, USA).

### miRNA microarray analysis

For the miRNA expression analyses, we used the miRNA microarray to process the samples (Super Biotek, China). A TaqMan Low Density Array v3.0 (Applied Biosystems) was used to detect and quantify up to 291 human miRNAs using an Applied Biosystems real-time instrument, according to the manufacturer's protocol, and the data were analysed further with Agilent GeneSpring GX 10 Microarray Data Analysis Software. In this study, differentially expressed miRNAs were selected according to the following criteria: the change fold was no less than 1.5-times for MHCC-97H exosomes vs. MHCC-97L exosomes and 5-times for MHCC-97L exosome vs. LO2 exosome; the *P* value was set at 0.05.

### Quantitative RT-PCR

Total RNA was isolated from the exosomal pellets. To validate miRNA expression, real-time quantitative PCR (qRT-PCR) was performed using SYBR Premix Dimer-Eraser kit (Takara Biotechnology, Dalian, China) on an ABI Prism 7300 HT Detection System (Applied Biosystems, CA, USA). U6 snRNA was used as an internal control. The primers for miR-665 and U6 were purchased from RiboBio (Guangzhou, China). The primers used are listed as follows: miR-665 (5′-GTCGTATCCAGTG CAGGGTCCGAGGTATTCGCAC TGGATACGACAG GGGC-3′), U6 (5′-GTCGTATCCAGTGCAGGGTCCG AGGTATTCGCACTGGATACGACAAAATA-3′). The relative gene expression values for the target miRNA were normalized to U6 snRNA and calculated using the 2^−ΔΔCT^ method.

### Lentivirus vector transfection

To obtain hepatoma cell-derived exosomes with downregulated miR-665, HCC cell lines (MHCC-97L and MHCC-97H) were seeded into a 6-well plate at a density of 5 × 10^5^ cells/well to a final volume of the culture solution of 2 mL. Cells were transfected at a multiplicity of infection (MOI) of 10, incubated at 37°C for 5 h and allowed to recover in fresh culture medium for 72 h at 37°C with 5% CO_2_, under the excitation wavelength HCC cells displayed green colour with green fluorescence microscopy.

### Cell viability assay

Cell viability was monitored using MTT assay. Generally, 5 × 10^3^ cells were allowed to grow in 96-well plates. After incubation with tumour-derived exosomes for 24 and 48 h, 20 μL of MTT solution (0.5%) was added to the medium for further incubation for 4 h. A total of 150 μL of DMSO was added to every well to dissolve the insoluble formazan product after removing the medium. The absorbance of the coloured solution was measured at 570 nm with a spectrophotometer. All experiments were performed in triplicate.

### Animal model

All animal procedures were performed according to national guidelines and approved by the Animal Care Ethics Committee of Nanjing Drum Tower Hospital. Fifteen male BALB/c nu/nu mice (4–6 weeks old, Laboratory Animal Center of Shanghai, Academy of Science). All mice received subcutaneous injections of SMMC-7721 cells in the right armpit (1 × 10^7^ cells in 200 μL of PBS per mouse). Fifteen mice were randomly divided into three groups (the control group, MHCC-97H-exosome group, and the MHCC-97H-exosome anti-miR665 group) when the tumours reached a volume of 50–100 mm^3^ (15 days after subcutaneous injections of tumour cells), subcutaneously injected with exosomes (100 μg of total protein, in the vicinity of the subcutaneous tumours), respectively, the control group was injected with equal volume PBS. The mice were examined every 2 days, and all mice were sacrificed by cervical dislocation under general anaesthesia with chloral hydrate (5%, 100 μL/10 g).

### Immunohistochemical

Formalin-fixed and paraffin-embedded subcutaneous tumour samples from nude mice were first cut into 5-μm-thick sections. Then, antigen retrieval was accomplished by deparaffinization, rehydration, and boiling in a microwave oven with citrated buffer. Hydrogen peroxide (3%) in PBS was used to block endogenous peroxidase activity, and BSA was used to block nonspecific staining. Sections were incubated with an anti-p-ERK polyclonal antibody (1: 200, bs-3292R, Bioss, Beijing, China) at 4°C overnight. The EliVision plus Kit (kit-9902, Maixin Biotech, China) was used to detect primary antibody followed by staining with DAB reagent and counterstaining with haematoxylin. Finally, the slides were imaged under the microscope (BX43, OLYMPUS, Japan).

### Statistics

All results, except the microRNA value, are described as the mean ± s.d. The relationships among miR-665 expression, clinicopathological factors and the *in vitro* assay data were analysed using Student's *t*-tests, X^2^-tests and ANOVA. Post-operative survival curves were plotted using the log-rank test. All differences were considered significant at the level of *P* < 0.05. All statistical analyses were performed using SPSS (Statistical Package for the Social Sciences) for Windows release 18.0 (SPSS Inc., Chicago, IL, USA).
